# Mapping the dynamic plant interactome: from *in vitro* assays to *in vivo* quantitative approaches

**DOI:** 10.1186/s13007-026-01571-0

**Published:** 2026-07-12

**Authors:** Muhammad Ans Hussain, Ameer Hamza Hafeez, Iqra Noor, Adeena Shakoor, Hammad Hussain, Fatemeh Gholizadeh, Hamza Sohail

**Affiliations:** 1 College of Horticulture and Forestry, Tarim University, Aral, 843300 China; 2 Xinjiang Production and Construction Corps Key Laboratory of Facility Agriculture, Tarim University, Aral, 843300 China; 3 College of Horticulture and Landscape Architecture, Yangzhou University, Yangzhou, 225009 China; 4 Department of Plant Physiology and Metabolomics, Agricultural Institute, HUN-REN Centre for Agricultural Research, Martonvásár, 2462 Hungary; 5 National Key Laboratory of Crop Genetic Improvement, Huazhong Agricultural University, Wuhan, 430070 China; 6 Hongshan Laboratory, Huazhong Agricultural University, Wuhan, 430070 China; 7 National Key Laboratory for Germplasm Innovation and Utilization of Horticultural Crops, National R&D Centre for Citrus Preservation, College of Horticulture and Forestry Science, Huazhong Agricultural University, Wuhan, P. R. China; 8 MOA Key Laboratory of Crop Ecophysiology and Farming System in the Middle Reaches of the Yangtze River, College of Plant Science and Technology, Huazhong Agricultural University, Wuhan, 430070 China; 9 Department of Plant Breeding and Genetics, Faculty of Agriculture, Akdeniz University, Antalya, Turkey

**Keywords:** Interactome mapping, Protein–protein interaction, Proximity labeling, Split-luciferase complementation, *In vivo* interactomics, Plant signaling

## Abstract

**Background:**

Protein–protein interactions underpin virtually all biological processes in plants, from signal transduction and immune responses to development and stress adaptation. Despite their fundamental importance, the plant interactome remains far from complete, and existing maps are systematically biased by the technical limitations inherent to conventional detection platforms.

**Main body:**

This review critically traces the evolution of protein–protein interaction methodologies, from foundational approaches to advance in vivo and quantitative platforms. Classical techniques such as the yeast two-hybrid system and in vitro pull-down assays operate outside physiological cellular environments and are poorly suited to capturing transient or condition-dependent interactions. Affinity purification coupled with mass spectrometry improves throughput but remains vulnerable to artifacts introduced during cell lysis and to the preferential loss of weak interactors. To address these shortcomings, proximity labeling with engineered biotin ligases, most notably the fast-acting variant TurboID, has emerged as a powerful strategy, enabling covalent biotinylation of protein neighborhoods within living cells prior to lysis and thereby preserving associations that conventional methods routinely miss. Because TurboID reports proximity rather than direct binding, its output requires downstream binary validation. Complementary *in planta* validation tools are equally critical for moving beyond discovery. Split-luciferase complementation assays based on the NanoLuciferase reporter provide exceptional sensitivity for binary interaction detection under native expression conditions, while Förster Resonance Energy Transfer measured through fluorescence lifetime imaging microscopy offers quantitative biophysical evidence of molecular proximity at endogenous expression levels, serving as a high-confidence validation approach. Emerging technologies, including high-throughput protein microarrays and optogenetically controlled dimerization systems, further expand the methodological repertoire available to the plant biology community.

**Conclusion:**

We propose a practical, integrative three-tier framework, combining proximity labeling for broad in vivo discovery, split-luciferase complementation for sensitive binary validation, and fluorescence lifetime imaging microscopy for quantitative confirmation, that systematically funnels candidate interactions from initial identification to physiologically rigorous verification. This framework synthesizes established best practices into a structured workflow applicable to mapping dynamic plant interactomes, though its optimal implementation will depend on the biological question, target protein class, and available resources.

## Introduction

The interactome, the comprehensive set of all protein–protein interactions (PPIs) within an organism, constitutes the functional scaffolding and circuitry of the cell. These interactions are a critical element of all biological systems [1]. In plants, PPIs govern every aspect of life. They form the signaling cascades that perceive and respond to environmental stresses [2], the receptor-ligand complexes that drive development, the multi-enzyme, metabolons, that channel metabolic flux [3], and the immune receptor complexes that defend against pathogens [4]. Consequently, mapping the plant interactome is not merely an academic cataloging exercise; it is a prerequisite for a systems-level understanding of plant biology and for the rational engineering of crop resilience and productivity. Despite this clear imperative, the plant interactome remains a largely uncharted territory. Compared to other model organisms like yeast and humans, the plant map is described as sparse, incomplete, and remarkably lagging [5, 6]. Furthermore, the existing data is known to be subject to noise and investigative biases [5], casting doubt on the physiological relevance of many reported interactions. We assert that this incomplete-map problem is not a simple function of insufficient effort, but rather a direct consequence of a deep, methodological mismatch between the tools used and the object of study.

A major limitation in mapping the plant interactome arises from its inherently dynamic nature [7]. Rather than a static network, protein–protein interactions are highly transient, weak, and tightly regulated in a spatio-temporal context, often restricted to specific subcellular compartments or specialized cell types such as guard cells. These features make many biologically critical interactions, particularly those involved in signaling, difficult to detect using conventional approaches [8]. Widely used methods such as the yeast two-hybrid (Y2H) system, although dominant in plant research, lack physiological relevance, as they are performed in a heterologous nuclear environment and fail to capture plant-specific contexts, including organellar localization, post-translational modifications, and temporal dynamics. Consequently, the current sparse plant interactome likely reflects methodological limitations rather than biological reality.

Throughout this review, three quantitative regimes are distinguished. In binary detection an interaction is recorded as present or absent (Y2H, BiFC, and NanoBiT in screening mode). In relative quantification, signal intensity or peptide ratio reports a change between conditions but not absolute abundance, as in label-free or labeled AP–MS [9], TurboID-based proximity labeling [10, 11], and the model-derived FRET–FLIM bound-donor fraction [12]. In absolute or biophysical quantification, a physical constant, dissociation constant (Kd), association rate, dissociation rate, or fluorescence lifetime, is recovered, as in SPR, MST [13, 14], isothermal titration calorimetry, three-cube Förster Resonance Energy Transfer (FRET) [15], and three-fluorophore FRET in plants [16]. Following established affinity conventions [17], we define affinity envelopes as tight (Kd in the low nM range), moderate (high nM to low µM), and weak or transient (mid-µM to mM); these bin edges are conventional and the practical detection envelope is method-dependent. The kinetic envelope (association and dissociation rates) is as informative as Kd, because transient interactions with sub-second residence times are captured by TurboID through kinetic covalent labeling yet are systematically missed by AP–MS even when Kd lies in the nM range [18]. We use this binary, relative, and absolute terminology consistently in the text and in Table 1.

**Table 1:** Comparative analysis of key protein–protein interaction methodologies discussed in this review

Method	In planta compatibility	Affinity sensitivity / Kd envelope	Quantification type	Stoichiometry capability	Structural / interface resolution	Material input	Assay duration	Throughput (approx.)	Capital cost (0–5 filled-circle scale)	Key reference(s)
Yeast two-hybrid (Y2H)	No (heterologous yeast nucleus)	None — binary; no Kd (qualitative)	Low — binary detection	None	None — interaction only	Yeast transformation; library scale	Days–weeks per screen	Highest — 10⁵–10⁶ pairs/screen	●○○○○ 1/5 ~ US$5–10 K	[10, 61, 68]
AP–MS / Co-IP	In vivo (complex) / ex vivo lysate	Moderate — no Kd; abundance-based	Moderate — label-free, SILAC, TMT, iBAQ	Moderate — inferable with SILAC + absolute bait quantitation	None — composition only	High (0.5–5 g tissue typical)	1–3 days per bait	Moderate — ~10² baits/study	●●●●● 5/5 MS access ~ US$300–600 K (shared)	[9, 54, 62, 63, 91]
Pull-down / Far-Western	In vitro (purified)	Moderate — semi-quantitative; direct binding	Moderate — binary / relative	None	None — direct pair; no interface map	Moderate–high purified protein	1–2 days	Low	●○○○○ 1/5 ~ US$1–5 K	[13, 94]
TurboID proximity labeling (TurboID-PL)	In vivo (key advantage)	Low — no Kd; captures transient (kinetic)	Moderate — relative enrichment (not stoichiometric)	None — relative only	Low — ~10 nm neighbourhood (not interface)	Moderate (transgenic/transient + biotin)	Labeling 10 min–3 h; MS days	Moderate — ~10¹–10² baits	●●●●● 5/5 MS-based platform + low reagent cost	[10, 53, 54]
BiFC	*In planta*	None — binary; no Kd	Low — binary detection (irreversible)	None	Low — proximity; slow maturation	Low (transient expression)	Hours–1 day	Moderate	●●●●○ 4/5 confocal ~ US$150–300 K (shared)	[64–66]
BRET / NanoBRET	*In planta*	High — semi-quantitative; relative proximity	High — ratiometric	High — apparent (ratiometric)	Low — <10 nm proximity; bulk/non-imaging	Low (transient expression)	Hours	High — plate reader	●●○○○ 2/5 plate reader ~ US$30–60 K	[94, 96, 97]
NanoLuc split-luciferase (NLuc-SLCA / NanoBiT)	*In planta*	High — high sensitivity; reversible	Moderate — luminescence (relative)	None — relative only	Low — reconstitution geometry	Low (transient expression)	Hours	High — plate reader	●●○○○ 2/5 plate reader / imager	[68–70]
Microscale thermophoresis (MST)	In vitro (purified or lysate)	Highest — Kd ~ pM–mM	Highest — absolute (Kd)	Moderate — indirect (titration)	None	Very low (5–10 µL)	Hours	Low	●●●○○ 3/5 ~ US$80–120 K	[13, 14, 89]
Surface plasmon resonance (SPR)	In vitro (purified)	Highest — Kd + kon/koff	Highest — absolute (Kd, kinetics)	Moderate — indirect (1:1 model)	None	Moderate purified protein	Hours	Low	●●●●○ 4/5 ~ US$150–250 K	[90]
Acceptor-photobleaching FRET (apFRET)	*In vivo / in planta*	High — proximity < 10 nm; semi-quantitative	High — relative (E per pixel)	High — apparent (with calibration)	High — <10 nm distance regime	Low (transient expression)	Hours (per field)	Low — per field	○○○○○ 0/5 no added capital over existing confocal	[91, 92]
FRET–FLIM	*In vivo / in planta*	High — proximity < 10 nm; concentration-independent	High — model-derived bound-donor fraction + apparent stoichiometry	Highest — bi-/multi-exponential fitting; 3-cube / 3-fluorophore	Highest — <10 nm; ternary-capable	Low (knock-in at endogenous loci sufficient)	Hours (per field)	Low — 1–5 pairs/instrument-day	●●●●○ 4/5 TCSPC ~ US$150–300 K incremental	[15, 16, 84]
Protein microarray	In vitro	Moderate — semi-quantitative; relative	Moderate — relative	None	None	High (large-scale purified protein)	Days	Highest — proteome-scale	●●●●○ 4/5 array platform	[12]
Optogenetics (CRY2–CIB1; phytochrome; channelrhodopsin)	*In vivo / in planta*	n/a	n/a	n/a	n/a	Low (transient/transgenic + light)	Minutes (light-gated)	n/a	●○○○○ 1/5 light source + confocal	[106, 107, 110]

*AP–MS* affinity purification–mass spectrometry, *apFRET* acceptor-photobleaching FRET, *BiFC* bimolecular fluorescence complementation, *BRET* bioluminescence resonance energy transfer, *Co-IP* co-immunoprecipitation, *FRET–FLIM* Förster resonance energy transfer–fluorescence lifetime imaging microscopy, *Kd* dissociation constant, *MST* microscale thermophoresis, *NanoBiT* NanoLuc Binary Technology, *NLuc-SLCA* NanoLuciferase split-luciferase complementation assay, *SPR* surface plasmon resonance, *TurboID-PL* TurboID proximity labelling,* Y2H* yeast two-hybrid

Table 1 compares the thirteen PPI methods discussed in this review across ten analytical and practical attributes

In the five capability columns (affinity/Kd, quantification, stoichiometry, interface/spatial resolution and throughput) the bold word gives the rank — None, Low, Moderate, High or Highest, assigned relative to the methods compared here — and the text after the dash states its factual basis. Capital cost is graded with a filled-circle scale from ○○○○○ (0/5; no additional dedicated capital beyond already available equipment) to ●●●●● (5/5; highest instrument/platform cost among the methods compared)

“n/a” denotes a metric that does not apply (optogenetics induces or releases interactions rather than measuring them)

To define the scope of this review more precisely, we focus on plant-compatible, dynamic, and *in planta* protein–protein interaction (PPI) workflows rather than providing a comprehensive survey of all interaction-mapping and structural biology approaches. Structure-based prediction, including AlphaFold-Multimer, can infer candidate interfaces [19]; cross-linking mass spectrometry (XL-MS) provides distance restraints [20]; co-fractionation mass spectrometry (CF-MS) captures native protein-complex organization in plant systems [21, 22]; and high-resolution methods such as X-ray crystallography, nuclear magnetic resonance (NMR), and cryo-electron microscopy (cryo-EM) resolve complex architecture. These approaches are powerful but address questions partly distinct from the focus of this review: they mainly clarify how complexes are organized, whereas we emphasize methods that determine when, where, and under which physiological conditions PPIs occur in living plant systems. We therefore treat structural approaches as complementary rather than omitted. Earlier plant-focused reviews provide broader context for in vivo PPI methods [23], large-scale interactome datasets [24], affinity- and complementation-based approaches [25], and chloroplast-centered plant–microbe interaction methods [26]. Building on this background, we emphasize a practical workflow linking in vivo discovery, *in planta* binary validation, and quantitative biophysical confirmation, using a consistent binary–relative–absolute framework throughout the review and Table 1.

This review critically examines the evolution of protein–protein interaction methodologies, from traditional in vitro and yeast-based systems to advanced in vivo, quantitative, and high-throughput approaches. We evaluate these methods based on their ability to address key challenges, including physiological relevance, detection of transient interactions, and avoidance of experimental artifacts. Emphasis is placed on the need for a paradigm shift toward *in planta*, context-aware, and quantitative strategies. Ultimately, we propose an integrated framework for best-practice interactome mapping that more accurately captures the dynamic complexity of plant cellular networks.

## Foundational approaches: strengths and enduring limitations

The plant interactomics toolbox has been built upon a set of foundational established core methods. While instrumental in identifying many key protein partners, these techniques are each beset by significant limitations. A critical re-assessment of these methods reveals that their limitations are not merely trivial caveats but systematic, methodological barriers that have, in large part, defined the boundaries of our current knowledge and are responsible for the sparse and noisy character of the interactome map [27].

### *In vitro* biochemical assays: probing direct physicality

*In vitro* biochemical assays provide a direct means to test whether two proteins can physically interact, independent of the complexity of the cellular environment. The most widely used approach is the pull-down assay, frequently implemented with a Glutathione S-transferase (GST) tag. In this method, the bait protein is expressed as a GST fusion, typically in *Escherichia coli* (*E.coli*), purified, and immobilized on glutathione-coated agarose beads [28]. The immobilized bait is then incubated with a prey sample, which may consist of a purified protein or a complex cell lysate [29]. After washing to remove unbound material, retained prey is detected, providing evidence for a direct interaction [28].

**Fig. 1:**
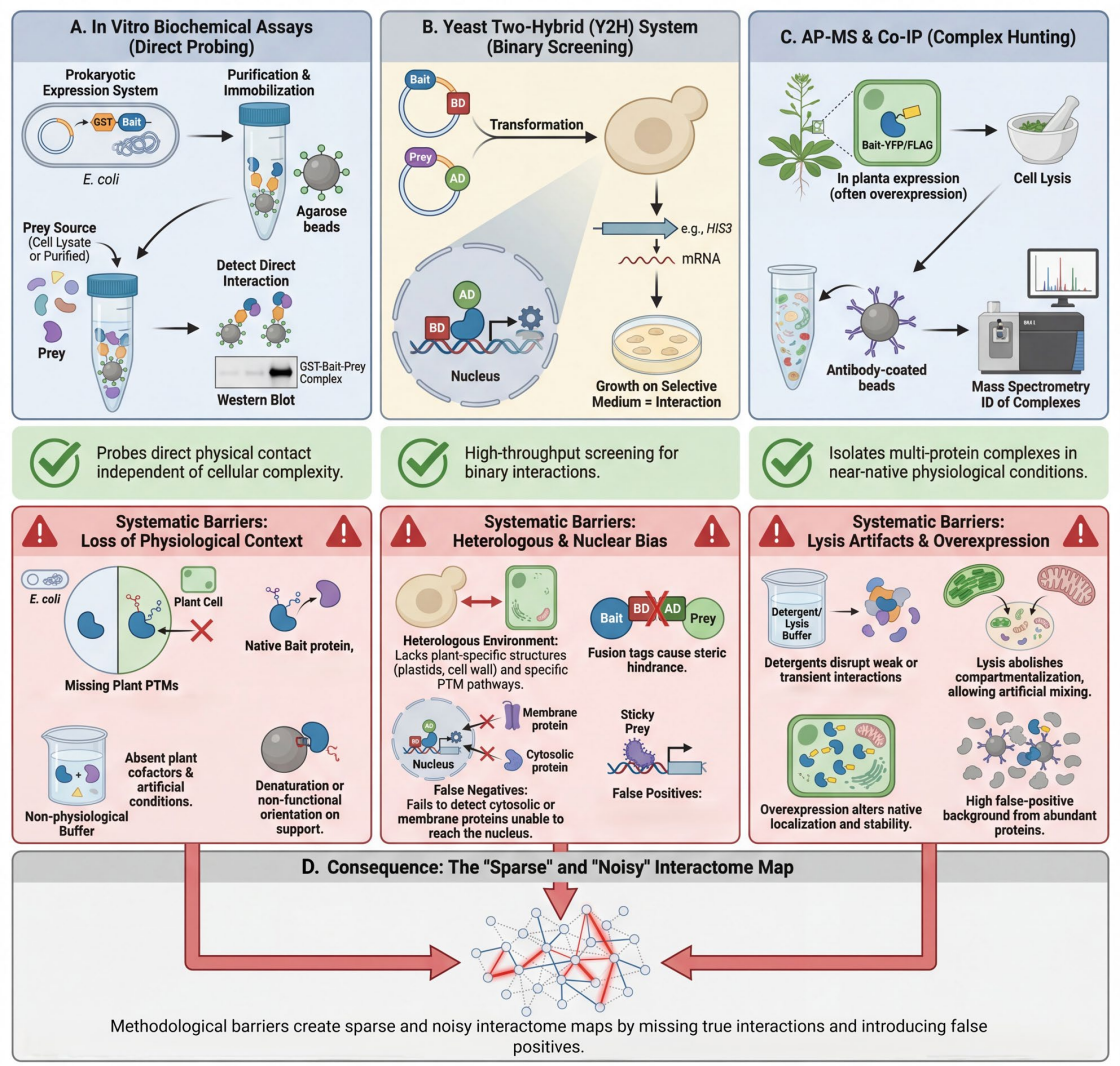
Foundational approaches in plant PPI mapping and their impact on interactome quality. The figure summarizes three core interactomics methodologies (**A**–**C **) commonly used in plant PPI studies and their collective impact on the quality of interactome maps (**D**).** A** In vitro biochemical assays (direct probing). Bait proteins (e.g., GST-tagged) are expressed in *E. coli*, purified, and immobilized on agarose beads. Prey material, either cell lysate or purified protein, is incubated with the bait-beads, and interactions are detected directly, for example by Western blotting of GST–bait–prey complexes. This approach provides direct evidence of physical contact between defined protein pairs, independent of cellular complexity, and thus supports high-resolution validation of individual PPIs. However, the artificial, non-plant conditions lead to loss of physiological context, including absence of plant-specific post-translational modifications (PTMs) and cofactors, potential denaturation or non-native orientation of immobilized proteins, and loss of native bait integrity during purification and immobilization. **B **Yeast two-hybrid (Y2H) system (binary screening). In this heterologous system, bait proteins are fused to a DNA-binding domain (BD) and prey proteins to an activation domain (AD); co-expression in yeast reconstitutes a functional transcription factor upon interaction, driving reporter gene expression (e.g., HIS3) and growth on selective medium. Y2H enables high-throughput, binary screening suitable for large-scale interactome mapping. Nonetheless, the nuclear-confined yeast environment lacks plant organelles and PTM pathways, and can introduce steric effects from fusion tags, compartmentalization bias for cytosolic and membrane proteins that fail to reach the nucleus, and false positives arising from promiscuous sticky proteins that activate reporters independently of specific PPIs. **C **Affinity purification–mass spectrometry (AP–MS) and co-immunoprecipitation (co-IP) (complex hunting). Epitope-tagged bait proteins (e.g., FLAG, YFP) are expressed *in planta*, typically under overexpression conditions. Following cell lysis, bait-containing complexes are captured with antibody-coated beads and analyzed by mass spectrometry. This strategy captures multi-protein complexes in a near-native plant cellular context and preserves aspects of physiological interactions. At the same time, detergent-based lysis can disrupt weak or transient interactions, loss of compartmentalization can promote artificial mixing of proteins, overexpression can alter protein localization and stability, and abundant cellular proteins frequently generate high background and false positives. **D **Consequence: a sparse and noisy interactome map. Accumulated biases across all three approaches yield interactome maps that are both sparse, owing to extensive false negatives where genuine interactions are missed, and noisy, owing to false positives where spurious interactions are reported. This is illustrated as an incompletely connected network with misassigned edges, emphasizing the need for integrative, multi-method strategies and rigorous downstream filtering to approach a more accurate, physiologically relevant plant interactome

A related strategy is Far-Western blotting. Here, prey proteins are first separated by SDS–PAGE and transferred to a membrane, analogous to a conventional Western blot [30]. Instead of probing with an antibody, the membrane is incubated with a purified, labeled bait protein [31], which binds directly to its interaction partner on the membrane and can then be detected. A standard protocol for this technique was described by Wu et al. (2007) [32] (Fig. 1A).

These in vitro assays are powerful for confirming direct physical contacts between proteins [33]. However, their principal limitation is the complete loss of physiological context. Bacterial expression systems generally do not reproduce the full plant-specific post-translational modification (PTM) landscape, such as phosphorylation or glycosylation patterns, that may be required for some interactions. Non-physiological buffer conditions, absence of plant cofactors, and potential denaturation during purification can all contribute to both false positives and false negatives. In addition, technical factors such as non–site-specific bioconjugation can result in random immobilization of proteins in non-functional orientations on solid supports, further complicating data interpretation [34].

### The Y2H system: a double-edged sword

First developed by Fields and Song in 1989 [35], the Y2H system has been widely used as a protein-fragment complementation assay (PCA) for high-throughput detection of PPIs in plants [36]. In the classical configuration, a bait protein is fused to the DNA-binding domain (BD) of a transcription factor and screened against a library of prey proteins fused to the activation domain (AD). Co-expression of these constructs in yeast allows interacting bait–prey pairs to reconstitute a functional transcription factor in the nucleus, thereby activating a reporter gene (e.g., HIS3) and enabling growth on selective medium [37].

Despite its utility, Y2H suffers from limitations that systematically bias plant interactome maps [24]. Because interactions are assayed in the yeast nucleus [37], the system operates in a heterologous environment that lacks plant-specific cellular structures (e.g., plastids, plant cell wall) and many PTM pathways and cofactors required for accurate regulation of protein function *in planta* [38]. This non-native context can alter protein localization, stability, and interaction specificity (Fig. 1B).

The assay also displays a high false-positive rate, generating numerous biologically irrelevant interactions [38]. These frequently arise from promiscuous or sticky proteins that non-specifically activate the reporter, necessitating additional validation steps, including switching bait and prey constructs to help eliminate false positives [33].

Conversely, Y2H exhibits a high false-negative rate and pronounced systematic biases. Because both fusion proteins must translocate to the nucleus, the method is poorly suited to detect interactions involving proteins that function predominantly in the cytosol, at the plasma membrane, or within organelles. This limitation motivated the development of alternative approaches, such as the split-ubiquitin system (SUS), specifically designed to detect interactions of membrane proteins [36]. Moreover, interactions that depend on plant-specific PTMs will not be recapitulated in yeast, and fusion of the relatively large BD and AD domains can sterically hinder proper folding or occlude native interaction surfaces of the proteins of interest [39].

### Affinity purification–mass spectrometry (AP–MS): hunting for complexes

To overcome the non-native environment inherent to Y2H, researchers have increasingly adopted affinity purification–mass spectrometry (AP–MS), including variants such as co-immunoprecipitation (Co-IP). In this approach, a bait protein is tagged (e.g., with YFP or a FLAG epitope) and expressed *in planta* [40]. A cell lysate is then prepared, and an antibody specific to the tag is used to pull down the bait together with its endogenously bound partners. The resulting protein complex is subsequently identified by mass spectrometry [7]. The principal advantage of AP–MS is its ability to isolate protein complexes under near-native physiological conditions, thereby better reflecting in vivo binding relationships [41]. In contrast to Y2H, which primarily detects binary interactions, AP–MS can reveal multi-protein complexes, providing a more comprehensive, systems-level view of interaction networks [42]. However, AP–MS is strongly influenced by lysis-dependent artifacts. The method critically depends on purifying complexes from a cell lysate, and the required extraction conditions introduce substantial biases [43]. The choice of lysis buffer and detergent composition can markedly affect the outcome, as the harsh conditions often needed to solubilize proteins and reduce non-specific background can disrupt weak or transient interactions, leading AP–MS to frequently miss the very interactions that underpin dynamic signaling pathways [44] (Fig. 1C). At the same time, lysis abolishes subcellular compartmentalization, allowing proteins from distinct organelles and compartments to mix artificially. This, together with promiscuous binding of highly abundant proteins to affinity matrices, contributes to a relatively high false-positive rate [45]. AP–MS workflows frequently rely on strong overexpression of the bait protein (e.g., under a 35 S promoter) to achieve sufficient recovery for MS analysis. Such overexpression may influence the bait’s physiological properties, including its localization, stability, and interaction profile, thereby promoting non-physiological associations [46].

Importantly, the limitations of these foundational techniques are often addressed through compensatory workflows rather than resolved. In the plant literature, in vivo Co-IP results are commonly validated with in vitro pull-down assays, pull-down hits are confirmed using Y2H [37] or pull-down is combined with split-luciferase assays [47]. While conceptually sound, this multi-method patching highlights the continuing need for a single, robust, in vivo technology that can capture physiologically relevant interactions while minimizing artifacts associated with cell lysis. Stoichiometric inference merits explicit treatment, as the quantification classes differ markedly in what they can and cannot deliver. Label-free AP–MS provides spectral counts that are semi-quantitative within a sample. The intensity-based absolute quantification index (iBAQ) normalizes MS1 intensity by the number of theoretical tryptic peptides per protein and yields within-sample abundance comparable across proteins in the same lysate [9]. SILAC (stable-isotope labeling by amino acids in cell culture) and TMT (tandem mass tags) provide cross-sample relative quantification by metabolic and isobaric labeling, respectively, while AQUA peptides and SRM/MRM with isotope-labeled standards provide absolute quantification per protein. Crucially, stoichiometric inference from AP–MS requires the bait to be quantified absolutely and all prey to be quantified on the same scale. TurboID, by contrast, yields relative enrichment rather than true stoichiometric ratios, because biotinylation efficiency depends on accessible-lysine density and labeling duration and must be stated as such [10, 48]. FRET–FLIM directly measures changes in donor fluorescence lifetime rather than a donor fraction. Apparent FRETing or bound-donor fractions can be estimated from appropriate bi- or multi-exponential decay models, but these fractions are model-derived parameters whose interpretation depends on the assumed decay model, fluorophore behavior, donor-acceptor stoichiometry, and underlying photophysics [49]. The three-cube FRET formalism decomposes the apparent signal into an efficiency (E) and a stoichiometry (S) parameter and returns intracellular stoichiometry when calibrated against a reference fusion of known composition [15], and three-fluorophore FRET extends this to ternary-complex stoichiometry in living plant cells [16].

## Proximity labeling: capturing the *in viv*o neighborhood

**Fig. 2:**
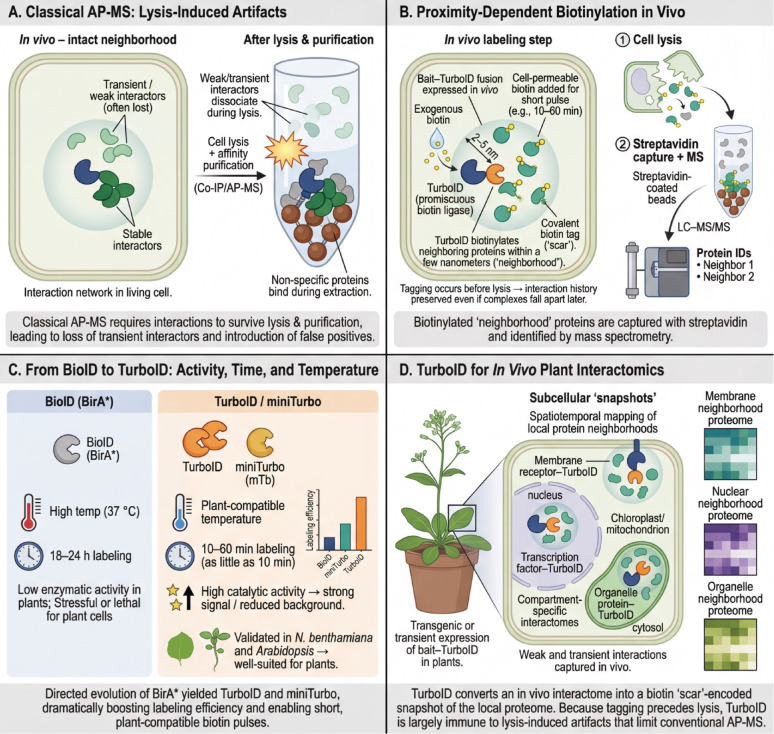
Comparison of classical AP–MS and TurboID-based proximity labeling for plant interactomics. This figure compares classical affinity purification–mass spectrometry (AP–MS) with proximity-dependent biotinylation, focusing on TurboID for plant interactome mapping. **A** Classical AP–MS – lysis-induced artifacts. This panel contrasts the native cellular environment with post-lysis purification. In the intact cell (oval), a dark blue bait protein is surrounded by green stable interactors and light green transient/weak interactors. After lysis and Co-IP/AP–MS, the bait retains mainly the stable interactors, while transient/weak interactors dissociate and are lost. Numerous small brown spheres represent non-specific proteins that bind during extraction, generating false positives. Together, this illustrates the difficulty of preserving transient interactions and the artifacts introduced by lysis in classical AP–MS workflows. **B** Proximity-dependent biotinylation in vivo – TurboID workflow. This panel depicts the TurboID approach in three steps. In intact cells, a bait–TurboID fusion (dark blue bait fused to an orange promiscuous biotin ligase) is expressed. Upon addition of cell-permeable biotin (light blue), TurboID biotinylates neighboring light green proteins within a ~ 2–5 nm radius, creating covalent black biotin scar tags before lysis and thereby preserving the interaction history. After labeling, cells are lysed, biotinylated proteins are captured on streptavidin-coated grey beads, and LC–MS/MS is used to identify neighbor proteins (Neighbor 1, Neighbor 2, etc.). This workflow recovers both stable and transient interactors. **C** From BioID to TurboID – activity, time, and temperature parameters. This panel compares BioID (BirA*) with TurboID and miniTurbo. BioID requires high temperature (37 °C) and long labeling periods (18–24 h), leading to low activity in plants and potential cellular stress or lethality. TurboID and miniTurbo (orange ligase symbols in a light orange section) function at plant-compatible temperatures with markedly shorter labeling times (10–60 min, as short as 10 min), higher catalytic activity, and improved labeling efficiency. A bar chart shows labeling efficiency for BioID (light blue bar), miniTurbo (green bar), and TurboID (orange bar), highlighting the superior performance of TurboID. TurboID and miniTurbo were generated by directed evolution of BirA*, optimized for short biotin pulses in plants and validated in N. benthamiana and Arabidopsis thaliana. **D** TurboID application in plant in vivo interactomics. This panel illustrates the use of TurboID in whole plants. A potted plant represents transgenic or transient expression of bait–TurboID fusion proteins. An inset cell shows compartment-specific interactome mapping with TurboID targeted to different subcellular locations: membrane receptors (green), nuclear transcription factors (purple), and organelle proteins in chloroplasts and mitochondria (dark green). Each construct consists of a blue bait protein fused to an orange TurboID. Colored checkered rectangles represent the resulting neighborhood proteomes from each compartment. The annotation emphasizes that TurboID converts in vivo interactomes into biotin scar-encoded snapshots, overcoming lysis-induced artifacts that limit conventional AP–MS and enabling a more accurate representation of plant protein interaction networks

The central challenge of AP–MS, the loss of transient interactors and the introduction of false positives during lysis, stemmed from a single problem: the interaction must survive cell disruption and purification. Proximity-Dependent Labeling (PL), a technique that covalently marks protein neighbors in vivo before lysis, represents the first truly transformative solution to this problem [44] (Fig. 2A).

### A leap in efficiency: from BioID to TurboID

The principle of PL, specifically proximity-dependent biotinylation, is a paradigm shift. In this method, a promiscuous biotin ligase enzyme is genetically fused to a bait protein of interest and expressed in vivo. The plant is then supplied with exogenous, cell-permeable biotin. The fused ligase promiscuously labels any protein that comes within its immediate vicinity (its zoom radius of a few nanometers) with a covalent biotin tag [50]. This tag acts as a permanent scar, an indelible record that the protein was, at one point, a close neighbor of the bait. After a short labeling period, the cell is lysed. At this point, it no longer matters if the transient interactions are broken; the biotin scar remains. All biotinylated proteins (the entire neighborhood) are then easily and robustly captured using streptavidin-coated beads and identified by mass spectrometry [50] (Fig. 2B). This approach prevents the disruption of spatial relationships and interaction networks. This powerful concept was first realized as BioID, which used a mutated *E. coli* biotin ligase, BirA*. However, BioID was a poor fit for plant biology. Its low enzymatic activity required long labeling times (> 18 h) and, crucially, a high temperature (37 °C), which is lethal or highly stressful for plants [51].

The key enabling breakthrough was the directed evolution of the BirA* enzyme to create hyper-efficient variants: TurboID and miniTurbo. These engineered ligases are dramatically more efficient, solving all the problems of BioID. A landmark 2019 study tested TurboID and miniTurbo in two plant model systems (*Nicotiana benthamiana* and *Arabidopsis*) and concluded they are well suited for use in plants. Their high efficiency allows for robust labeling at room temperature in as little as 10 min, compared to the > 18 h for BioID [52] (Fig. 2C, D). A valid TurboID experiment depends on three categories of control. First, spatial mock controls, expression of an unfused TurboID, or a compartment-targeted free fluorescent-protein–TurboID fusion, establish the baseline of non-specific background biotinylation in the same subcellular compartment as the bait; without them, abundant proteins and the endogenously biotinylated carboxylase subunits (BCCP, MCCa/MCCb, and the propionyl-CoA carboxylase subunits) accumulate as false positives [10, 53]. Second, biotin supply must be optimized, because endogenous biotin pools and tissue permeability vary markedly between plant organs: for *Arabidopsis* root cultures, 50 µM exogenous biotin for approximately 3 h is a common starting point [52], whereas for transiently transformed *N. benthamiana* leaves, higher concentrations (up to ~ 1 mM) over 1–3 h are typical, reflecting a thicker cuticle and different endogenous biotin levels; a systematic benchmarking of BioID, BioID2, TurboID, and miniTurbo across *Arabidopsis*, *Nicotiana*, and *Marchantia* provides the practical envelope [53]. Third, quantitative cut-offs — enrichment over the mock control, statistical significance across at least three biological replicates, and orthogonal validation by Y2H, BiFC, or NanoBiT — are needed to separate genuine neighbors from sticky or abundant background. The effective labeling radius of TurboID is approximately 10 nm, calibrated against the nuclear pore complex [54], and longer pulses sample a measurably larger neighborhood [48]; the labeled set therefore contains direct partners, indirect complex members, and bystanders that TurboID alone cannot distinguish, which is why orthogonal binary or biophysical validation is essential.

### TurboID as a transformative tool for plant proteomics

TurboID represents a major advance in plant proteomics, providing an in vivo proximity-labeling strategy that combines the strengths of affinity purification–mass spectrometry with greatly reduced susceptibility to lysis-induced artifacts. This powerful proximity labeling approach is particularly well suited to address core challenges in plant interactomics [55]. A central advantage of TurboID is its ability to capture weak and transient protein interactions directly in living cells, which frequently fail to be detected by classical Co-IP/AP–MS workflows [41]. The engineered enzyme exhibits much-improved catalytic activity, leading to higher labeling efficiency and a dramatically increased signal-to-noise ratio [56]. Because labeling is driven by the addition of biotin, which is generally non-toxic and can be applied over short, controlled time windows, TurboID enables spatiotemporal mapping of local protein neighborhoods in vivo [52, 56]. The readout of a TurboID experiment is therefore not merely a list of binders but a snapshot of the subcellular proteome surrounding the bait protein at a defined moment. Finally, covalent tagging occurs in vivo before cell disruption, so subsequent lysis and harsh purification conditions do not erase information on transient or compartment-specific associations. As a result, TurboID is largely immune to the lysis artifacts that limit conventional AP–MS-based interaction mapping [57].

### Landmark applications in plant biology

The impact of TurboID in plant biology is exemplified by its rapid adoption to address long-standing questions that were poorly served by classical interaction assays. In the first report of TurboID use in plants, the authors applied TurboID-based proximity labeling to the N nucleotide-binding leucine-rich repeat (NLR) immune receptor. Using N as bait, they identified the E3 ubiquitin ligase UBR7 as a regulator of N-mediated immunity and showed that UBR7 directly interacts with N and mediates its turnover during resistance to tobacco mosaic virus [57, 58]. This study demonstrated that TurboID can resolve transient regulatory interactions within immune signaling pathways that had been difficult to capture using conventional methods. Mair et al. (2019) used TurboID to achieve cell-type- and compartment-specific proteomic profiling [52]. By fusing TurboID to a nuclear localization signal, they selectively labeled nuclear proteins in rare plant cell types, including guard cells, and subsequently identified these proteins by mass spectrometry. This work established that TurboID can define local protein neighborhoods within specific subcellular compartments of defined cell types *in planta*.

TurboID has also been deployed to dissect epitranscriptomic complexes. In one study, a TurboID-based proximity labeling–mass spectrometry screen using the m⁶A methyltransferase component FIP37 as bait was employed as a simple and rapid technique for efficient screening of interacting protein candidates. The approach recovered known components of the m⁶A methyltransferase complex, including MTA (the m⁶A methyltransferase catalytic subunit), MTB (its homolog and obligate complex partner), VIR (Virilizer, a scaffolding subunit homologous to the metazoan VIRMA/KIAA1429), and HAKAI (an E3 ubiquitin-ligase component of the m⁶A writer complex), as well as additional candidate interactors [59, 60]. Together, these applications illustrate that TurboID enables in vivo identification of transient interactions, cell-type- and compartment-specific proteomes, and the composition of regulatory complexes, providing functional insights that extend beyond the reach of traditional protein–protein interaction methods.

It is important to position TurboID accurately as a discovery method rather than as a universal solution for protein–protein interaction mapping [10, 48]. Proximity-dependent biotinylation is a kinetic-capture approach, not a binary-affinity assay: the engineered ligase generates a reactive biotinoyl-AMP intermediate that labels accessible lysines within an approximately 10 nm envelope [54]. Because the reactive intermediate can diffuse a short distance before hydrolysis, the labeled set may include direct binding partners, indirect complex members, and bystander proteins that share the same bait microenvironment [48, 54]. In addition, labeling efficiency can be influenced by bait expression level, subcellular compartment, accessible lysine density, biotin exposure time, and local background biotinylation [10, 52, 53]. For these reasons, plant TurboID studies consistently require appropriate spatial controls, quantitative enrichment over background, biological replication, and downstream validation of selected candidates [10, 53]. Thus, TurboID is best interpreted as a powerful in vivo neighborhood-mapping approach that generates a high-confidence candidate set, provided that background contaminants are controlled and candidate confidence is supported by appropriate statistical or probabilistic filtering [62, 63]. Direct binding, affinity, stoichiometry, and biophysical mechanism should then be established through subsequent validation assays suited to the specific biological question. A broader cross-method comparison is therefore provided later, after the relevant validation and biophysical approaches have been introduced, and is summarized in Table 1.

## *In vivo* binary assays: the evolution of protein-fragment complementation

While TurboID is a powerful tool for discovery, its output is a neighborhood and does not necessarily distinguish direct, binary interactions from indirect, proximal neighbors [53]. Therefore, validating hits from a TurboID screen—or any high-throughput screen—remains a critical step. For decades, this validation role was filled by Y2H. As discussed, Y2H has limitations as a validator due to its non-physiological environment. This created a need for *in planta* binary assays that are sensitive, robust, and minimally disruptive.

Historically, the first widely adopted *in planta* protein-fragment complementation tool was bimolecular fluorescence complementation (BiFC), in which two non-fluorescent halves of a fluorescent protein, typically YFP or mVenus, are fused to candidate partners so that their association reconstitutes a chromophore that can be imaged directly in plant cells [64, 65]. BiFC has well-documented properties that define its appropriate use [66]: chromophore maturation is slow, approximately 30 min to several hours; once formed, the reconstituted fluorophore is essentially irreversible, which makes BiFC excellent for spatial localization of stable interactions but unsuitable for following dynamic association and dissociation; and the fragments can self-assemble when overexpressed, so titration and competition controls are required. Split-luciferase complementation provides a complementary capability that BiFC lacks: because fragment association is reversible, the luminescent signal tracks the real-time assembly and disassembly of a complex [67]. This reversibility is engineered explicitly in the NanoBiT system, where the small fragment (SmBiT) was selected for low intrinsic affinity, with an intrinsic LgBiT–SmBiT Kd of approximately 190 µM, so that complementation reflects the bait–prey interaction rather than spontaneous fragment association [68, 69]. Consequently, split-luciferase and NanoBiT assays are suited to monitoring ligand- or stress-induced changes in interaction and to quantitative kinetic measurements that BiFC cannot resolve. Regarding the interpretation of the signal itself, a reconstituted luminescent signal indicates that complementation has occurred and therefore that the tagged proteins are in close proximity in a correct relative orientation, but signal magnitude is not a direct readout of a single interaction. The measured intensity convolves at least three variables: the expression levels of the two fusions, the probability and occupancy of the interaction, related to its Kd under the prevailing concentrations, and the geometric compatibility of the reconstituted fragments. Therefore, intensity should be interpreted as a relative, semi-quantitative measure across matched conditions rather than as an absolute count of interacting molecules or a direct dissociation constant.

### Split-luciferase complementation assays (SLCAs)

**Fig. 3:**
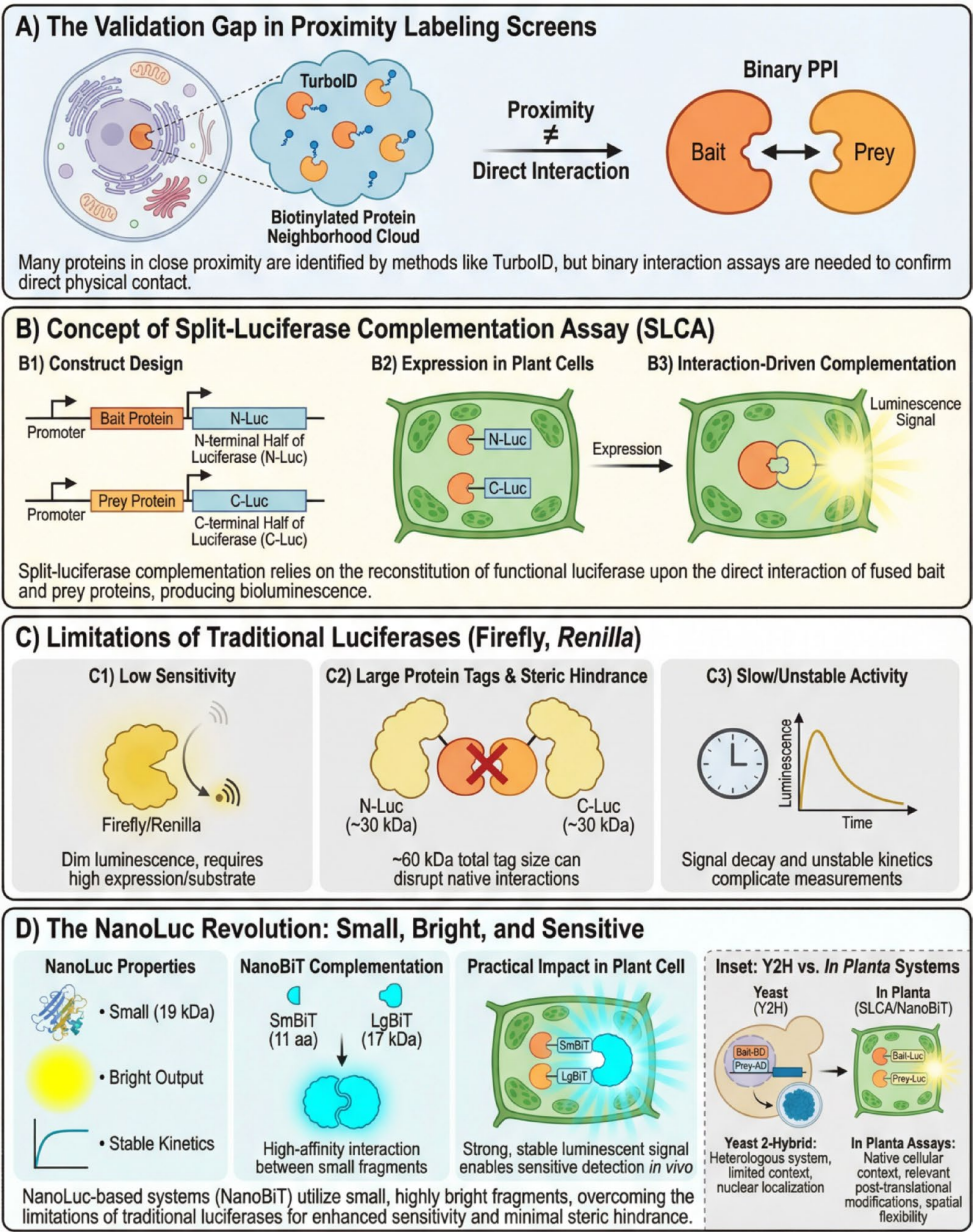
Evolution of in vivo binary interaction assays: From split-luciferase to NanoLuc-based complementation. This figure illustrates the technological progression and methodological necessity of split-luciferase assays in plant biology, structured into four panels (**A**–**D**). **A** The validation gap in proximity labeling screens. This panel highlights the critical distinction between physical proximity and direct interaction. Proximity labeling techniques, such as TurboID, are depicted on the left, where a bait protein (orange) fused to a blue TurboID enzyme biotinylates proximal proteins within a specific radius, creating a Biotinylated Protein Neighborhood Cloud (light blue halo) inside a plant cell. The central arrow emphasizes that Proximity ≠ Direct Interaction, as many proteins identified in the cloud are neighbors but may not physically touch the bait. To confirm genuine binary PPI, a validation step is required; the right side illustrates a true binary PPI where orange bait and prey proteins directly bind, distinguishing them from mere neighbors. (**B**) Concept of split-luciferase complementation assay (SLCA). This panel breaks down the mechanism of SLCA into three sequential stages. First, construct design involves two expression cassettes where the bait and prey proteins are fused to the N-Luc and C-terminal (C-Luc) halves of luciferase, respectively. Second, these constructs are introduced into plant cells (green background) where the fusions float separately in the cytosol. Third, interaction-driven complementation occurs when the bait and prey interact physically, bringing the N-Luc and C-Luc fragments into close proximity to reconstitute a functional enzyme. In the presence of luciferin, this produces a measurable bioluminescence signal (yellow glow), confirming the interaction. **C** Limitations of traditional luciferases (Firefly, Renilla). This panel critiques older luciferase systems by identifying three specific barriers to accurate data: low sensitivity, depicted as dim luminescence (faint yellow glow) requiring high expression levels; large protein tags (~ 60 kDa combined) causing steric hindrance (indicated by a red X) that physically blocks native interactions; and slow or unstable activity, illustrated by a clock icon and a signal decay graph that complicates time-course measurements. **D** The NanoLuc revolution: small, bright, and sensitive. This panel presents the superior attributes of the NanoLuc-based NanoBiT system. The full NanoLuc enzyme is highlighted as small (19 kDa), capable of bright output (yellow sun icon), and possessing stable kinetics (flat line graph). The system uses optimized fragments—SmBiT (11 amino acids) and LgBiT (17 kDa)—that exhibit high affinity only when brought together. A plant cell is shown emitting a strong, stable luminescent signal (bright cyan/blue glow), demonstrating high sensitivity suitable for in vivo detection. An inset comparison contrasts the yeast two-hybrid (Y2H) system (heterologous, nuclear-limited) with in planta assays, emphasizing that SLCA/NanoBiT occurs in the native cellular context, allowing for relevant post-translational modifications and spatial flexibility. This figure collectively argues for the adoption of NanoLuc-based tools to bridge the gap between high-throughput screens and precise binary interaction mapping

Building on this progression from irreversible fluorescent complementation to reversible luminescent complementation, split-luciferase complementation assays (SLCAs) provide a sensitive *in planta* binary-validation approach for testing candidate protein–protein interactions [70]. In SLCAs, two proteins of interest are fused to complementary, non-functional fragments of a luciferase enzyme, such as an N-terminal fragment (Nluc-N) and a C-terminal fragment (Nluc-C). When these fusion constructs are co-expressed in plant cells, interaction between the candidate partners brings the luciferase fragments into proximity, reconstituting an active enzyme. Upon addition of the luciferin substrate, restored luciferase activity produces a luminescent signal that reports on interaction-dependent complementation [71]. These assays can be performed in plant cellular systems, including *Arabidopsis* protoplasts and transiently transformed *N. benthamiana* leaves, and are particularly useful for evaluating candidate interactions under matched expression and treatment conditions. Two features make SLCAs especially useful for *in planta* validation: first, they are performed in plant cells, providing a more physiologically relevant context for plant protein–protein interactions; second, because plants lack endogenous bioluminescence, luciferase-based readouts exhibit high signal-to-noise ratios and enable sensitive detection of interacting pairs. The ability to monitor complex assembly quantitatively and in real time makes SLCA a powerful approach for *in planta* validation of protein–protein interactions [72] (Fig. 3A, B).

### The NanoLuciferase (NLuc) revolution

The evolution of split luciferase complementation assays (SLCA) parallels the development of BioID and TurboID. The SLCA concept was powerful, but traditional luciferases derived from Firefly (*Photinus pyralis*) or *Renilla* exhibited important limitations [73]. In particular, signals from Firefly- and *Renilla*-based assays were often very difficult to detect, restricting sensitivity for weak interactions. In addition, as with other protein-fragment complementation assays, fusion of large reporter proteins (e.g., green fluorescent protein, GFP, or the ~ 60 kDa firefly luciferase) to proteins of interest can disrupt endogenous interactions, for example through steric hindrance or perturbation of protein folding. Protein engineering again transformed the field with the development of NLuc, a small (19 kDa) luciferase engineered from the deep-sea shrimp *Oplophorus gracilirostris*, which mitigates both low sensitivity and tag-induced artifacts [71]. NLuc exhibits extremely bright bioluminescence, improving the signal-to-noise ratio and enabling sensitive detection of interactions that are poorly resolved with earlier luciferases. Its small size is an additional advantage, as it may reduce steric interference and make NLuc fusions less likely to perturb native interaction networks [71] (Fig. 3C, D).

This transition from large, relatively dim luciferases to a small, bright reporter parallels the shift from slow, temperature-restricted BioID to the more efficient TurboID. In both cases, protein engineering has yielded tools that minimize experimental artifacts while maximizing sensitivity, thereby improving the fidelity of in vivo interaction measurements. In current practice, NLuc-based SLCA (often referred to as NanoBiT) is widely used for robust *in planta* validation of binary protein–protein interactions and is frequently employed as a downstream complement or alternative to Y2H.

## Biophysical validation: quantitative FRET–FLIM at native expression levels

While NLuc-SLCA provides excellent *in planta* validation of a binary interaction, it typically still relies on overexpression and reports a yes/no (or quantitative “how much”) answer. The methodological gold standard, the technique that provides the highest possible degree of physiological evidence, can prove an interaction in vivo, in a specific cell, at native concentrations, and in a quantitative, biophysical manner. This standard is met by FRET–FLIM.

### Principles of FRET–FLIM

Förster resonance energy transfer (FRET) is a biophysical, distance-dependent transfer of excitation energy between two fluorophores [74]. In a typical FRET experiment, a donor fluorophore (e.g., GFP) is fused to one protein of interest, and an acceptor fluorophore (e.g., red fluorescent protein, RFP) is fused to its putative interaction partner. When the two proteins interact and bring the fluorophores into nanoscale proximity, typically below ~ 10 nm, excitation of the donor by a laser can result in non-radiative transfer of energy to the acceptor. This process quenches donor fluorescence and concomitantly induces acceptor emission [75]. FRET was formulated quantitatively by Förster in 1948 [78]. The transfer efficiency follows E = 1 / (1 + (r/R_0_)⁶), where r is the donor–acceptor distance and R_0_ is the Förster radius, defined as the distance at which the probability of energy transfer is 50%. FRET efficiency is mainly governed by three interdependent parameters: the spectral overlap between donor emission and acceptor excitation, the distance between donor and acceptor, and the relative orientation of their transition dipoles. The more favorable these parameters are, the higher the FRET efficiency [79].

For fluorescent-protein pairs used in plant and live-cell FRET studies, the Förster radius typically lies in the nanometer range, for example CFP–YFP R_0_ approximately 4.9 nm [79], GFP–mCherry R_0_ approximately 5.1 nm, and mTurquoise2–mVenus R_0_ approximately 5.6 nm [80]. For this reason, the term interaction as used here in the FRET context denotes molecular co-occurrence within a FRET-detectable nanoscale distance regime, typically below ~ 10 nm, rather than direct physical contact per se. Where direct binding is the load-bearing claim, it should therefore be corroborated by an orthogonal method such as NanoBiT, co-crystallization, or AlphaFold-Multimer. Because R_0_ depends on the refractive index, donor quantum yield, and donor–acceptor spectral overlap, *in planta* calibration of the chosen pair is advisable [81].

Conventional FRET assays rely on changes in fluorescence intensity, also known as sensitized emission, but intensity-based readouts are highly susceptible to artifacts arising from fluorophore concentration, spectral bleed-through, direct acceptor excitation, photobleaching, and donor–acceptor stoichiometry. Quantitative FRET imaging is also subject to well-recognized photophysical constraints that apply across organisms [15, 82], including photophysical heterogeneity of fluorescent proteins and unfavorable dipole orientation. The orientation factor κ² ranges from 0 to 4, with the isotropic average of 2/3 used by convention in distance calculations [83]. Under free rotation, distance estimates are relatively robust, whereas restricted rotation can introduce larger uncertainty; selecting a FRET pair with well-characterized spectra and an appropriate R_0_ is therefore essential [80, 84]. Three-fluorophore FRET configurations extend the approach to ternary complexes in living plant cells, providing direct evidence for higher-order assemblies that binary assays cannot access [16].

**Fig. 4:**
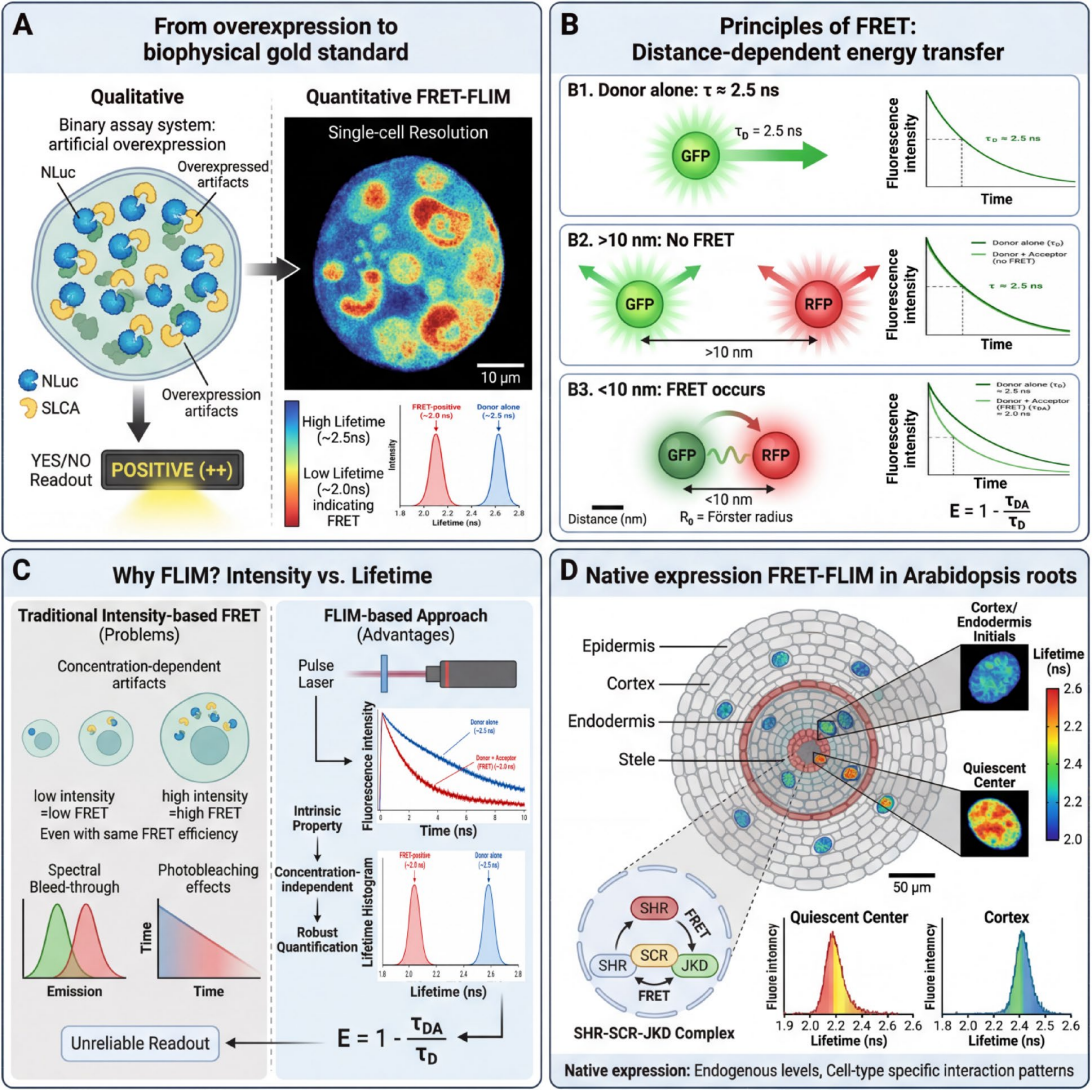
Biophysical validation of plant protein interactions at native expression levels using quantitative FRET–FLIM. This figure highlights how fluorescence lifetime–based FRET analysis has emerged as a gold-standard biophysical approach for validating protein–protein interactions (PPIs) in living plant cells. **A** From overexpression-based assays to quantitative biophysics. Conventional binary interaction assays, such as NLuc–SLCA, rely on artificial overexpression and provide only qualitative YES/NO outcomes, often confounded by stoichiometric imbalance and cellular artifacts. In contrast, quantitative FRET–FLIM enables direct measurement of nanoscale molecular proximity in vivo by visualizing fluorescence lifetimes at single-cell resolution. This shift from intensity-based detection to lifetime-based quantification provides a more accurate and physiologically grounded assessment of PPIs. **B** Biophysical principles of Förster Resonance Energy Transfer (FRET). FRET occurs when an excited donor fluorophore transfers energy non-radiatively to an acceptor within ~ 10 nm—a distance comparable to macromolecular complexes. GFP exhibits a donor lifetime (~ 2.5 ns) when alone (B1), which remains unchanged when the acceptor is beyond the Förster radius (B2). In close proximity (B3), FRET accelerates donor fluorescence decay and shortens donor lifetime (e.g., ~ 2.0 ns), providing a quantitative optical signature of interaction strength and efficiency. **C** Why FLIM? Advantages of lifetime-based quantification. Intensity-based FRET approaches are susceptible to concentration dependence, photobleaching, detector nonlinearity, and spectral cross-talk, complicating quantitative interpretation. FLIM overcomes these limitations by fitting fluorescence intensity decay to extract donor lifetime, an intrinsic parameter independent of fluorophore abundance or excitation intensity. Thus, reduced donor lifetime directly reports FRET efficiency (E = 1 – τ_DA/τ_D), providing a robust, quantitative, and concentration-independent measure of PPI strength in vivo. **D** Native-expression FRET–FLIM in Arabidopsis roots. By deploying functional fusion proteins driven by endogenous promoters, FRET–FLIM enables biophysical validation of PPIs at native expression levels. In the Arabidopsis root, lifetime mapping of nuclei reveals cell-type–specific interaction signatures for the SHR–SCR–JKD complex, a key regulatory module in radial patterning. Distinct lifetime shifts in the cortex/endodermis initials and quiescent center demonstrate that FRET–defined interactions remain detectable without overexpression, establishing the physiological relevance and spatial specificity of these molecular complexes in planta

The modern, more robust implementation couples FRET with fluorescence lifetime imaging microscopy (FLIM). FLIM measures the fluorescence lifetime of the donor fluorophore, the average time it remains in the excited state before photon emission, which is an intrinsic physical property largely independent of fluorophore concentration [76, 77]. In the absence of FRET, the donor exhibits its characteristic lifetime, for example ~ 2.5 ns for GFP. When the donor-tagged protein is brought into FRET-permissive proximity with the acceptor-tagged partner, energy transfer provides an additional de-excitation pathway for the donor and thereby reduces its fluorescence lifetime, for example to ~ 2.0 ns. This FRET-induced decrease in donor lifetime provides a quantitative and physically well-defined measure of nanoscale proximity, and thus strong evidence consistent with protein–protein interaction in living cells. Lifetime-based FRET–FLIM is therefore considered superior to intensity-based FRET for the analysis of complex interaction scenarios, including ternary protein complex formation [76, 77] (Fig. 4A, B).

It is important, however, to define the quantitative output precisely. FRET–FLIM directly measures changes in donor fluorescence lifetime rather than a donor fraction. Apparent FRETing or bound-donor fractions can be estimated from appropriate bi- or multi-exponential decay models [49]. However, these fractions are model-derived parameters whose interpretation depends on the assumed decay model, fluorophore behavior, donor–acceptor stoichiometry, and underlying photophysics [84–86]. Although the donor lifetime is largely independent of donor concentration, the donor: acceptor stoichiometry can influence the recovered apparent bound-donor fraction and FRET efficiency, an effect that the three-cube FRET formalism corrects explicitly and that has been reviewed for plant systems [85]. A landmark *in planta* demonstration of FRET–FLIM at native expression resolved cell-type-specific interactions in *Arabidopsis* roots [87], and for quantitative interpretation the advantage of lifetime data over a simple yes/no readout is that donor lifetime changes provide a continuous, concentration-independent readout of molecular proximity [84–86].

### The “native expression” breakthrough

Despite its strengths, FRET–FLIM, like AP–MS and SLCA, was historically implemented with proteins expressed from strong constitutive promoters, leaving unresolved the possibility that observed interactions were artifacts of overexpression rather than physiological events [3]. The landmark study by Long et al. (2017) provided the first rigorous demonstration of FRET–FLIM at native protein levels in plants [87]. Using in vivo FRET–FLIM, the authors examined interactions among the *Arabidopsis* root cell fate regulators SHORT-ROOT (SHR), SCARECROW (SCR), and JACKDAW (JKD). Crucially, they showed that FRET–FLIM could be performed with fully functional, fluorescently tagged versions of these regulators expressed from their own promoters at endogenous expression levels. The approach was sufficiently sensitive to resolve cell-type-specific interactions within individual nuclei of the living root and revealed that these transcription factors assemble into higher-order complexes in a spatially restricted manner.

This study, together with a subsequent 2018 work that optimized FRET–FLIM labeling conditions for detection at native expression levels, established FRET–FLIM as a definitive method for assessing physiological relevance. It demonstrated that protein–protein interactions can be validated in vivo, in situ, within defined cell types and at native protein concentrations [8, 87] (Fig. 4C, D).

### Application case study: visualizing in vivo metabolic complexes

FRET–FLIM provides more than a simple yes/no assessment of interaction; it can also reveal how a protein complex is organized and functions in vivo. A 2019 *Plant Cell* study by Mucha et al. (2019) illustrates this capability in the context of camalexin biosynthesis, a key plant defense pathway [88]. Although the enzymes involved, including several P450s such as CYP71A12 and CYP71B15, had been identified, their spatial organization was unclear. By combining Co-IP with FRET–FLIM, the authors showed that these enzymes are not merely diffusely distributed in the endoplasmic reticulum but instead physically associate to form a metabolon, a stable multi-enzyme complex. This arrangement is hypothesized to enhance metabolic efficiency by channeling reactive intermediates directly between active sites, thereby limiting diffusion and reducing self-toxicity [88].

This work highlights the higher-order potential of FRET–FLIM, extending its utility beyond interactome mapping into the realm of in vivo structural and mechanistic analysis. A similar conceptual advance was noted by Long et al. (2017), who reported that FRET–FLIM lifetime distributions reflected conformational changes within SHR/SCR/JKD complexes [87]. These conformational states were proposed to differentially regulate target gene expression and specify distinct cell fates. Thus, FRET–FLIM not only detects molecular proximity but can discriminate between inactive and active conformations of multi-protein assemblies, providing a level of mechanistic detail that is difficult to achieve with other interaction assays.

Although FRET–FLIM is biophysically powerful, it faces an important accessibility barrier: it requires specialized time-correlated single-photon-counting (TCSPC) detectors and instrumental expertise that are absent in many plant molecular biology laboratories [86]. Importantly, the methods discussed below should be viewed as complementary or question-specific supporting approaches, not as direct replacements for FRET–FLIM. MST measures the temperature-induced redistribution of fluorescently labeled molecules and can return Kd values across approximately pM to mM using small sample volumes [13, 14, 89], while SPR measures real-time mass binding at a sensor surface and returns kinetic on- and off-rates and Kd for purified analytes [90]. However, MST and SPR are in vitro methods and cannot determine whether an interaction occurs in the correct subcellular compartment, cell type, expression context, or native physiological environment of a living plant cell. Similarly, acceptor-photobleaching FRET (apFRET) can be performed on a standard confocal microscope and provides an accessible in-cell readout of FRET efficiency [91–93], while BRET and NanoBRET use a luciferase donor to reduce autofluorescence background and can be implemented with standard plate-reader infrastructure [94, 95]. BRET-family assays have also been demonstrated in plant cells [96, 97]. Nevertheless, apFRET and BRET/NanoBRET do not provide the same lifetime-based, spatially resolved, concentration-independent evidence as FRET–FLIM [76, 84–86]. Thus, when the biological question requires direct, in vivo, cell-resolved, lifetime-based evidence of nanoscale proximity, FRET–FLIM remains the most appropriate validation method. These companion methods are therefore best considered useful for screening, orthogonal support, purified-protein quantification, or laboratories without TCSPC infrastructure, provided that their limitations are clearly recognized [13, 14, 89–97].

## Emerging frontiers: high-throughput and optogenetic control

**Fig. 5:**
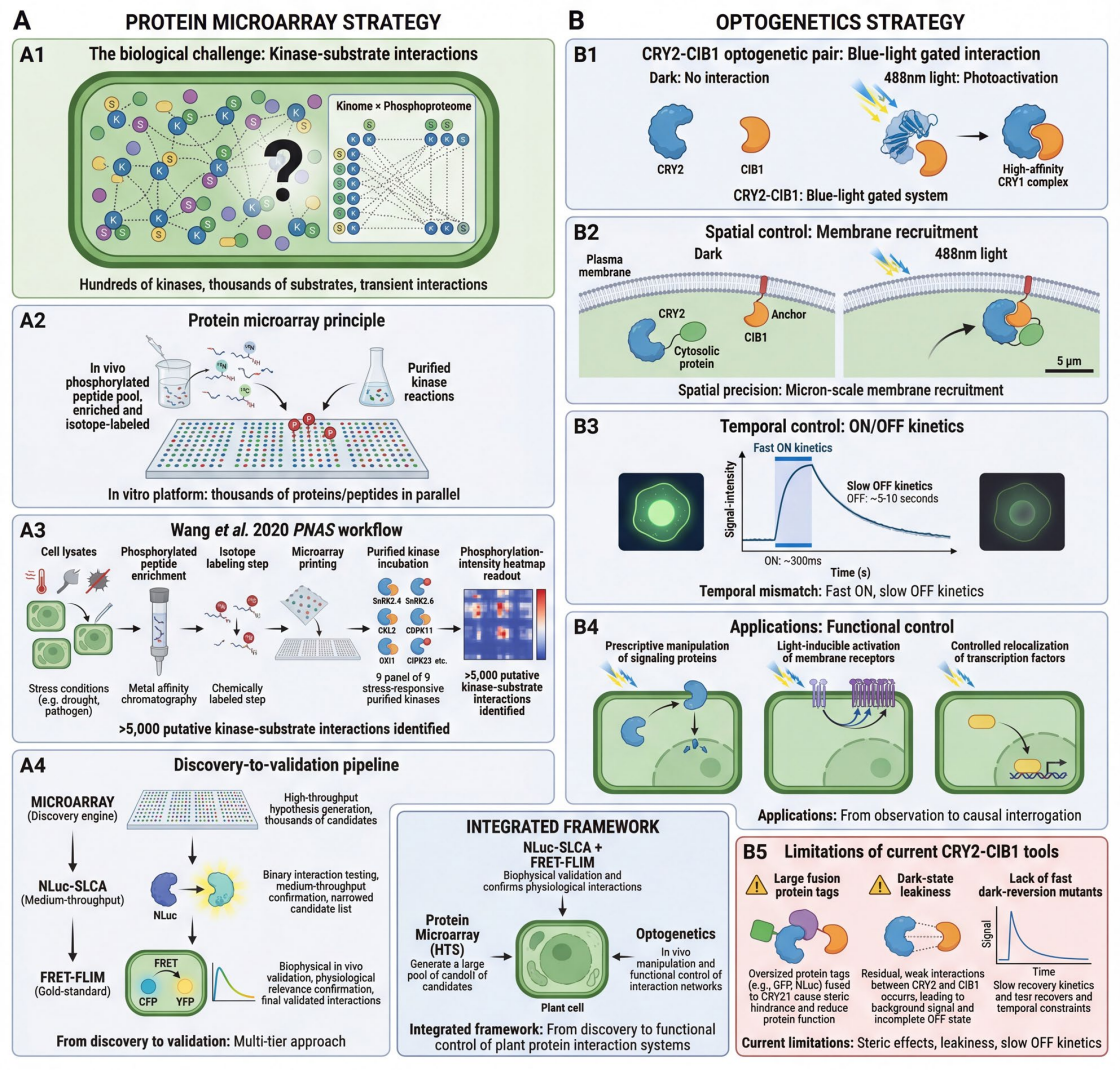
Emerging frontiers in plant protein interaction biology. This figure summarizes two rapidly advancing methodological frameworks, protein microarray–based biochemical screening and optogenetic control systems, that are transforming how plant protein interaction networks are identified, validated, and functionally interrogated. **A** Protein microarray strategy: high-throughput discovery of kinase–substrate interactions. Panels A1–A4 outline how protein microarrays have enabled systematic mapping of plant kinase signaling pathways, which are notoriously difficult to capture due to their transient and often low-affinity nature. Modern microarray platforms integrate phosphopeptide enrichment, isotope labeling, and on-array kinase assays to survey thousands of substrates in parallel, providing proteome-scale views of kinase specificity. These datasets feed into a tiered validation pipeline in which putative interactions identified biochemically are subsequently assessed using medium-throughput assays (e.g., NLuc-SLCA) and then confirmed via biophysical measurements such as FRET–FLIM. Together, these tools collectively enable a transition from broad discovery to high-resolution validation at physiological expression levels. **B **Optogenetic strategy: spatiotemporal control of protein interactions in vivo. Panels B1–B5 illustrate how plant-adapted optogenetic modules, particularly the blue-light–responsive CRY2–CIB1 system, allow researchers to manipulate protein interactions with precise spatial and temporal resolution. Light-induced CRY2–CIB1 binding enables controlled protein recruitment to membranes, acute modulation of signaling nodes, and programmable relocalization of transcription factors. This provides a powerful complement to observational interaction assays by enabling causal testing of how protein interactions influence downstream cellular behaviors. Despite their utility, current optogenetic tools still face practical constraints such as slow dark-state reversion, limited dynamic range, and potential steric effects introduced by fusion tags. Integrated perspective. Together, the biochemical breadth of protein microarrays, the biophysical rigor of interaction validation assays, and the functional precision of optogenetics represent a converging toolkit for plant signaling research. This integrated pipeline enables researchers not only to map interaction networks at scale but also to probe their physiological relevance and causative roles within living plant cells—an emerging frontier in plant systems biology

As the tools for in vivo observation and validation have matured, two new frontiers have emerged. The first seeks to apply in vitro screening at a massive, high-throughput scale to rapidly generate hypotheses. The second represents a conceptual leap from describing the interactome to controlling it (Fig. 5).

### High-throughput screening: protein microarrays

Protein discovery remains a major challenge, particularly at the level of entire protein families. Protein microarrays have emerged as a powerful high-throughput screening (HTS) technology to address this need, and are especially well suited to the kinase–substrate problem [98, 99]. Kinase-substrate interactions are paradigmatic transient, low-affinity interactions that are central to signaling yet difficult to capture, and defining the full substrate spectrum for the hundreds of kinases in the plant genome remains a major unresolved task [100].

A 2020 *PNAS* study by Wang et al. introduced a novel HTS strategy based on isotope-labeled in vitro phosphorylation reactions [101]. In this approach, in vivo phosphorylated peptides were used as substrate pools and immobilized on microarrays, which were then probed with purified kinases. This yielded a proteome-wide map of kinase targets, identifying more than 5,000 putative kinase–substrate pairs for nine stress-responsive protein kinases. This resource highlights the power of microarrays for high-throughput in vitro discovery. However, all > 5,000 interactions remain putative and in vitro; they constitute a large hypothesis set that must be validated *in planta* using complementary methods such as NLuc-based SLCA and FRET–FLIM [101] (Fig. 5A).

### Spatio-temporal control: optogenetics

All methods discussed thus far are primarily descriptive, designed to observe protein interactions that are already occurring. Optogenetics provides a complementary, prescriptive strategy by enabling light-controlled induction of interactions, thereby allowing direct tests of causality between complex assembly and phenotype. The most widely used optogenetic dimerization system in plants is the *Arabidopsis*-derived CRY2–CIB1 module [102, 103].

In darkness, the photoreceptor cryptochrome 2 (CRY2) and its binding partner CIB1 do not interact. Upon blue light illumination, CRY2 undergoes a conformational change that exposes a CIB1-binding site, resulting in strong, rapidly inducible association [103]. This light-gated interaction can be harnessed in synthetic biology applications by fusing CRY2 to a cytosolic protein of interest and CIB1 to a defined cellular location, such as the plasma membrane. In the absence of light, the protein remains cytosolic; localized illumination with a 488 nm laser can then acutely recruit the CRY2-fusion to the CIB1-labeled membrane region in real time, enabling precise spatial and temporal control over protein localization and allowing direct assessment of the consequences of relocalization. This strategy effectively co-opts the plant’s endogenous light-sensing machinery for experimental control of cellular processes [103, 104].

Despite its utility, the CRY2–CIB1 system has notable limitations, including the relatively large size of the fusion tags, a measurable dark baseline interaction that leads to leakiness in the absence of light, and the lack of robust dark-reversion mutants, which results in slow dissociation and delayed return to the pre-activation state after light removal [105] (Fig. 5B). Beyond the blue-light CRY2–CIB1 dimerizer, several optogenetic systems have been demonstrated in plants, and plant-specific first reports deserve explicit acknowledgement. A red and far-red phytochrome-based gene-expression switch was first established in the moss *Physcomitrella patens* [106], and an analogous system tailored for use under ambient white light was subsequently engineered for *Arabidopsis* [107]. Reversible red-light control modules developed in other systems [108] and the broader plant flavoprotein photoreceptor toolkit [109] provide further plant-relevant options. Channelrhodopsin-based control has also been shown in plants: channelrhodopsin-2 (ChR2; activated near 470 nm) and the red-shifted variant ChrimsonR (activated near 590 nm), expressed in *Dionaea muscipula* and *Arabidopsis*, drive light-gated cation flux that elicits action-potential-like depolarizations and downstream signalings [109, 110], extending the toolkit from gene-expression control to membrane-physiology control. Together these spectrally distinct tools enable multiplexed control and bring plant-native photoreceptors into the plant synthetic-biology repertoire.

## Synthesizing a modern toolkit: an integrated three-tier workflow

The historical development of plant interactomics, spanning high-throughput but artifact-prone AP–MS approaches and low-throughput yet spatially resolved microscopy, underscores a central principle: no single method can simultaneously achieve optimal sensitivity, specificity, and physiological relevance [24] (Table 1). The recent maturation of TurboID-based proximity labeling, NanoLuciferase split complementation, and FRET–FLIM now enables these complementary strategies to be combined into a unified, rationally ordered workflow.

Here, we propose a hierarchical three-tier pipeline that functions as a funnel, progressively refining the candidate space from a broad set of putative interactors to a small cohort of high-confidence, physiologically validated partners. Each tier exploits a distinct biophysical strength, kinetic capture, structural stringency, and thermodynamic realism, respectively, to systematically filter the proteome.

### Tier 1: discovery via in vivo kinetic capture

The workflow begins with unbiased discovery using TurboID-based proximity labeling as a functional replacement for conventional AP–MS. The key advantage of this strategy is a conceptual shift from equilibrium maintenance to kinetic capture [41].

In traditional AP–MS, the recovery of protein complexes relies on preserving non-covalent interactions during dilution, detergent exposure, and mechanical stress associated with cell lysis. Under these conditions, interactions characterized by rapid dissociation kinetics (high k_off_) are preferentially lost, leading to systematic underrepresentation of transient signaling complexes.

TurboID, an engineered biotin ligase with accelerated catalytic kinetics, labels proteins within a defined spatial radius on the timescale of minutes [47]. This reaction generates a covalent and durable biochemical record of the local protein environment, often faster than the dissociation time of transient complexes. By effectively snap-freezing weak or short-lived interactions into stable biotin adducts prior to cellular disruption, Tier 1 yields a high-sensitivity inclusion set, the proxitome, that captures both stable and transient interactors that are frequently missed by classical biochemical purification.

### Tier 2: validation via structural filtering

Although proximity labeling maximizes sensitivity, its spatial resolution is constrained by the effective labeling radius of TurboID (~ 10 nm). As a result, datasets invariably include bystander proteins that are co-localized within the same subcellular microenvironment but do not physically contact the bait. Distinguishing direct binding partners from these non-interacting neighbors requires a stringent structural filter [111].

NanoLuciferase-based Split Luciferase Complementation Assays (NLuc–SLCA) provide such a filter by enforcing physical proximity and correct orientation of bait and prey to reconstitute luminescent activity. In contrast to proximity-labeling approaches, complementation only occurs when the two fusion partners form a stable complex that aligns the split fragments correctly.

Mechanistically, NLuc is advantageous over earlier split reporters (e.g., Firefly luciferase or YFP) due to the small size of its fragment tags (~ 1.3 kDa and ~ 18 kDa) [71]. The reduced steric bulk minimizes perturbation of native protein structure and interaction interfaces, thereby decreasing tag-induced artifacts. Consequently, a positive NLuc–SLCA signal more reliably reflects a native physical interaction rather than forced proximity or artificial oligomerization. Filtering the Tier 1 proxitome through this binary assay removes most indirect neighbors and retains a refined set of high-probability direct binding partners.

### Tier 3: confirmation under physiological thermodynamic constraints

The final tier addresses physiological relevance. Both proximity labeling (typically relying on ectopic expression) and split-luciferase assays (often driven by strong promoters) can promote non-physiological interactions via mass-action effects, where elevated local protein concentrations increase the likelihood of stochastic collisions. Distinguishing genuine in vivo interactions from such overexpression artifacts requires a method that operates at endogenous expression levels and is insensitive to fluorophore concentration.

FRET–FLIM fulfills these criteria and serves as the gold standard for thermodynamically grounded validation. By monitoring fluorescence lifetime rather than intensity, FLIM decouples measurement from fluorophore abundance and photobleaching [75]. It quantifies changes in the donor’s excited-state lifetime (τ), which decreases only when non-radiative energy transfer occurs to an acceptor within Förster distance (< 10 nm) via dipole–dipole coupling.

Importantly, the sensitivity of contemporary FLIM systems allows measurements using proteins expressed from their native genomic loci and endogenous promoters. Under these conditions, a significant reduction in donor lifetime provides direct biophysical evidence that the proteins interact at native stoichiometries and physiological concentrations. Moreover, the imaging-based nature of FLIM preserves spatial information, pinpointing interactions within their subcellular and tissue context, for example, within a metabolon localized on the ER membrane or a transcription factor complex in a specific root cell nucleus [88]. This final tier thus anchors molecular interaction data in the actual cellular environment, completing the transition from broad discovery to rigorously validated, physiologically meaningful protein–protein interactions.

## Conclusion and future perspectives

This review has traced the evolution of plant interactomics from foundational in vitro assays to quantitative in vivo biophysical methods. Collectively, these approaches converge on a central conclusion: no single technique is sufficient to comprehensively define the plant interactome [7]. Y2H screens alone are prone to substantial false-positive and false-negative rates, affinity purification–mass spectrometry (AP–MS) is intrinsically biased against weak and transient interactions, and even TurboID-based proximity labeling primarily reports a local neighborhood of proximal proteins rather than strictly direct binders [41, 52], This has necessitated a transition from single-method studies to compensatory workflows in which the output of one assay (e.g., Y2H) is cross-validated with others (e.g., co-immunoprecipitation) [41]. The emergence of newer technologies, TurboID, NanoLuciferase-based split complementation assays (NLuc-SLCA), and FRET–FLIM, now permits a more systematic and integrated framework. Together, these tools can support a structured, three-tier workflow that leverages their complementary strengths to generate a robust, multilayered, and physiologically relevant view of a protein’s interaction network.

In a modern workflow, discovery is centered on defining the in vivo interaction neighborhood of a protein of interest (POI). TurboID-based proximity labeling is the preferred discovery platform [112], effectively supplanting AP–MS in this role because covalent tagging in living cells preserves weak and transient partners that are often lost during lysis and purification [113]. The result is a high-confidence map of the POI’s subcellular neighborhood. For specialized questions, such as systematic identification of kinase substrates, this discovery phase can be complemented by high-throughput in vitro protein microarray screening, as demonstrated for kinase–substrate mapping [114]. Validation of direct, binary interactions *in planta* is then required to refine the candidate set. Classical Y2H, constrained by a non-physiological nuclear environment and yeast-specific artifacts, is poorly suited to this role in plants. NLuc-based SLCA provides a more appropriate standard: the exceptional brightness and small size of NanoLuciferase enable sensitive detection of binary interactions with reduced risk of tag-induced perturbation, making NLuc-SLCA well suited for rapid *in planta* confirmation of direct interactors.

For the most critical interactions emerging from this validation step, a final layer of biophysical and physiological proof is provided by FRET–FLIM [115]. This technique offers quantitative, lifetime-based evidence that an interaction occurs in vivo, within the correct subcellular compartment [116], and, when implemented with endogenously expressed, fluorescently tagged proteins, at native expression levels, thereby avoiding overexpression artifacts [117]. Beyond simple detection of proximity, FRET–FLIM uniquely yields mechanistic insight into complex organization and conformational changes, enabling direct linkage between interaction states, target gene regulation, and cell fate decisions [118].

A workflow integrating TurboID-based discovery, NLuc-SLCA validation, and FRET–FLIM confirmation provides a robust framework for future plant interactome studies by combining complementary strengths across discovery, validation, and mechanistic characterization. Continued technological development will further enhance the utility of these approaches. For example, optogenetic systems would benefit from dimerizers exhibiting lower dark-state activity and faster dissociation kinetics than the current CRY2–CIB1 module [119]. Equally important is the application of these methodologies beyond *Arabidopsis* and *N. benthamiana* to economically important crop species, enabling the characterization of dynamic interaction networks associated with agronomically relevant traits. As methodological limitations continue to diminish, the field is increasingly equipped to resolve the complex and dynamic protein interaction networks that underpin plant growth, development, and environmental responses.

## Data Availability

No datasets were generated or analysed during the current study.
